# Resistance to anti-EGFR therapies in metastatic colorectal cancer: underlying mechanisms and reversal strategies

**DOI:** 10.1186/s13046-021-02130-2

**Published:** 2021-10-18

**Authors:** Jing Zhou, Qing Ji, Qi Li

**Affiliations:** 1 Department of Medical Oncology and Cancer Institute, Shuguang Hospital, Shanghai University of Traditional Chinese Medicine, Shanghai, 201203 China; 2 Academy of Integrative Medicine, Shanghai University of Traditional Chinese Medicine, Shanghai, 201203 China

**Keywords:** Metastatic colorectal cancer, Anti-epidermal growth factor receptor targeted therapies, Drug resistance, Reversal strategies

## Abstract

Cetuximab and panitumumab are monoclonal antibodies (mAbs) against epidermal growth factor receptor (EGFR) that are effective agents for metastatic colorectal cancer (mCRC). Cetuximab can prolong survival by 8.2 months in *RAS* wild-type (WT) mCRC patients. Unfortunately, resistance to targeted therapy impairs clinical use and efficiency. The mechanisms of resistance refer to intrinsic and extrinsic alterations of tumours. Multiple therapeutic strategies have been investigated extensively to overcome resistance to anti-EGFR mAbs. The intrinsic mechanisms include EGFR ligand overexpression, EGFR alteration, *RAS/RAF/PI3K* gene mutations, ERBB2/MET/IGF-1R activation, metabolic remodelling, microsatellite instability and autophagy. For intrinsic mechanisms, therapies mainly cover the following: new EGFR-targeted inhibitors, a combination of multitargeted inhibitors, and metabolic regulators. In addition, new cytotoxic drugs and small molecule compounds increase the efficiency of cetuximab. Extrinsic alterations mainly disrupt the tumour microenvironment, specifically immune cells, cancer-associated fibroblasts (CAFs) and angiogenesis. The directions include the modification or activation of immune cells and suppression of CAFs and anti-VEGFR agents. In this review, we focus on the mechanisms of resistance to anti-EGFR monoclonal antibodies (anti-EGFR mAbs) and discuss diverse approaches to reverse resistance to this therapy in hopes of identifying more mCRC treatment possibilities.

## Background

Metastatic colorectal cancer (mCRC) accounts for almost half of the newly diagnosed colorectal cancer cases and is associated with poor prognosis [1]. Epidermal growth factor receptor (EGFR) is a key factor in cellular proliferation, differentiation and survival [2], which drives the use of EGFR-targeted therapy in malignancy treatment [3]. The advent of cetuximab and panitumumab, two monoclonal antibodies (mAbs) directly targeting EGFR, can prolong survival for 10–20% of mCRC patients [4]. According to the CRYSTAL trial, the application of cetuximab and FOLFIRI in first-line treatment can reduce the risk of progression by 15% and increase overall survival (OS) by 8.2 months in patients who have *KRAS* WT mCRC compared with patients taking FOLFIRI alone [5].

Although treatment with anti-EGFR monoclonal antibodies (anti-EGFR mAbs) and chemotherapy has a large effect on mCRC, its clinical application is limited because of drug resistance. The clinical benefit in responders treated with anti-EGFR mAbs has been shown to only last 8–10 months [6, 7]. As treatment progresses, approximately 80% of responders develop drug resistance [8]. The mechanisms of resistance to anti-EGFR mAbs have been elucidated previously. Gene mutations downstream of the EGFR signalling pathway, including RAS/RAF/MEK and PI3K/AKT/mTOR, significantly contribute to drug resistance [9–11]. The activation of compensatory feedback loops of EGFR, such as erb-b2 receptor tyrosine kinase 2 (ERBB2), MET and insulin-like growth factor 1 receptor (IGF-1R), has been shown to interfere with EGFR inhibitor treatment [12–14]. In recent years, the intrinsic mechanisms of metabolism, autophagy [15], cancer stem cells (CSCs) [16] and epithelial-to-mesenchymal transition (EMT )[17] have also been confirmed to be correlated with poor progression despite anti-EGFR mAb treatment. Extrinsic alterations of tumours may appear during treatment with cetuximab and panitumumab [18]. Currently, it is believed that microenvironment remodelling can reduce the cytotoxicity of anti-EGFR mAbs by impairing antibody-dependent cellular cytotoxicity (ADCC) and secreting growth factors [19, 20].

Consequently, strategies to reverse resistance to anti-EGFR mAbs have been explored in experimental studies and clinical trials. These strategies include different aspects, such as new EGFR-targeted inhibitors, combinations of multitargeted inhibitors, metabolic regulators, immune therapy and new cytotoxic drugs. Here, we review the mechanisms underlying resistance to anti-EGFR mAbs and discuss the current studies on improving the efficiency of targeted therapy, increasing the number of available mCRC therapies.

## Intrinsic mechanisms of resistance to targeted therapy and related strategies

**Fig. 1:**
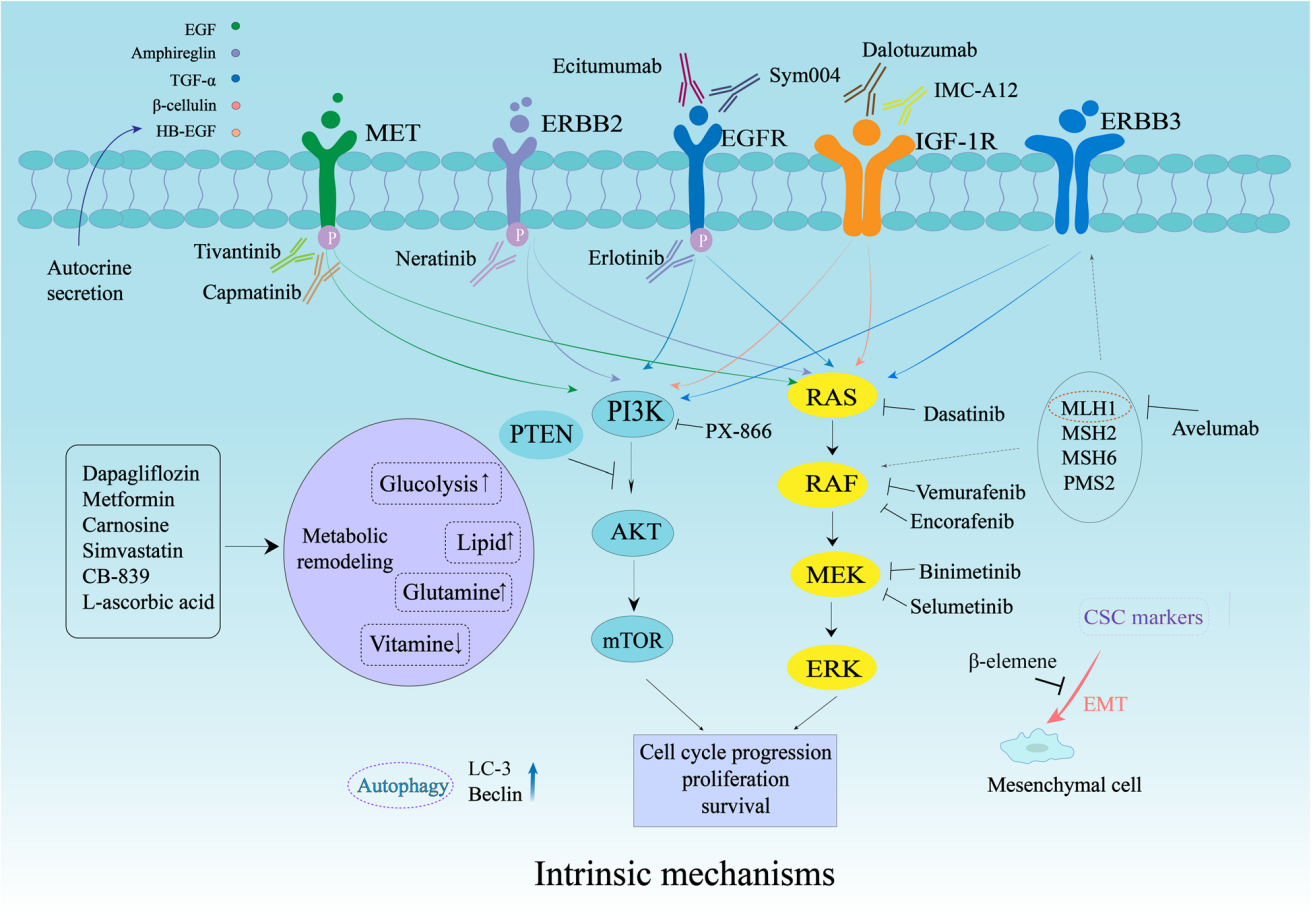
Intrinsic mechanisms of resistance to anti-EGFR mAbs in metastatic colorectal cancer. The intrinsic mechanisms include abnormal activation of oncogenic signalling pathways, aberrant gene expression, metabolic disorders, increased autophagy function and cancer stem cells. For example, genomic alterations and proteic phosphorylation induce activation of the RAS/RAF/MEK/ERK and PI3K/AKT/mTOR cascades. ERBB2/MET amplification and abnormal IGF-1R activation stimulate compensatory feedback loop signalling of EGFR. The phenotype shift of cancer stem cells (CSCs) into epithelial-to-mesenchymal transition (EMT) contributes to therapy resistance. Glycolysis, lipid synthesis, fatty acid oxidation and vitamin deficiency in cancer cells also reduced the efficiency of EGFR-targeted therapy. The agents for specific points are also shown in the figure. Abbreviations: CSC, cancer stem cell; EMT, epithelial-to-mesenchymal transition; PI3K, phosphoinositide 3-kinase; IGF-1R, insulin-like growth Factor 1 receptor

Intrinsic alterations of tumours greatly contribute to resistance to anti-EGFR targeted therapy. Known intrinsic mechanisms are genetic mutations inducing EGFR and compensatory feedback loop signalling activation. Recently, metabolic remodelling, CSCs and EMT have also been confirmed to promote resistance to targeted therapy (Fig. 1). Accordingly, different strategies have been used to reverse the resistance: (*i*) development of new EGFR targeted inhibitors, (*ii*) combination of anti-EGFR mAbs with multitargeted inhibitors, (*iii*) metabolic regulators and (*iv*) new cytotoxic drugs (Tables 1 and 2).

**Table 1:** Strategies to reverse resistance to anti-EGFR mAbs in clinical trials

Therapy	Target	Agents	Setting	Species	Subpopulation	Treatment regimen	Efficiency	Reference
New anti-EGFR mAbs	EGFR S468R	necitumumab	Phase II	mCRC	Unselected	Necitumumab plus mFOLFOX6	mPFS:10.0m; mOS:22.5m	[21]
	EGFR ECD	Sym004	Phase I	mCRC	*KRAS* WT	Sym004 or investigator’s choice	mOS: 12.8m VS 7.3m	[22]
	EGFR-TK	Erlotinib	Phase II	mCRC	*KRAS* WT	Erlotinib+ cetuximab	ORR:42%; mPFS:5.6m	[23]
RAS inhibitors	RAS	Dasatinib	Phase IB/II	mCRC	*KRAS* mutation	Dasatinib + FOLFOX +cetuximab	Not reached	[24]
	BRAF	Vemurafenib	Phase IB	mCRC	*BRAF* V600E mutation	Vemurafenib + Irinotecan + cetuximab	ORR:35%; mPFS:7.7m	[25]
RAF inhibitors			Phase II	mCRC	Unselected	Vemurafenib+ cetuximab VS cetuximab	ORR:0 VS 4%; mPFS3.7 VS 4.5m; mOS:7.1m VS 9.3m	[26]
		Encorafenib	Phase III	mCRC	*BRAF* V600E mutation	Encorafenib + binimetinib + cetuximab VS cetuximab chemotherapy	ORR: 26% VS 2%, mOS: 9.0m VS 5.4m	[27, 28]
MEK inhibitors	MEK	Binimetinib	Phase III	mCRC	*BRAF* V600E mutation	Encorafenib + binimetinib + cetuximab VS cetuximab chemotherapy	ORR: 26% VS 2%, mOS: 9.0m VS 5.4m	[28]
		Selumetinib	Phase I	mCRC	*KRAS* mutation	Selumetinib + cetuximab	Not reached	[29, 30]
ERBB2 inhibitors	ERBB2	Neratinib	Phase II	mCRC	*KRAS, NRAS, BRAF, PIK3CA* WT	Neratinib + cetuximab	Not reached	[31]
PI3K inhibitors	PI3K	PX-866	Phase II	mCRC	*KRAS* WT	PX-866 + cetuximab VS cetuximab	mPFS:59d VS 104d; mOS:266d VS 333d	[32]
MET inhibitors	MET	Tivantinib	Phase II	mCRC	*KRAS* mutation	Tivantinib + cetuximab	ORR: 9.8%, mPFS: 2.6m,mOS:9.2m	[33]
		Capmatinib	Phase II	mCRC	*MET* amplification	Capmatinib + gefitinib	ORR: 47%	[34]
IGF-1R inhibitors	IGF-1R	Dalotuzumab	Phase II/III	mCRC	*KRAS* WT	Dalotuzumab + Irinotecan + cetuximab VS placebo + Irinotecan + cetuximab	mPFS: 5.4m VS 5.6m;mOS:11.6 VS 14.0m	[35]
		IMC-A12	Phase II	mCRC	Unselected	IMC-A12 + cetuximab VS IMC-A12	Non response	[36]
Metabolic regulators	SGLT2	Dapagliflozin	Case report	mCRC	SGLT2+	Dapagliflozin + cetuximab	CEA dropped and tumor regression	[37]
Immune checkpoint inhibitors	PD-L1	Avelumab	Phase II	mCRC	*RAS* WT	Avelumab + cetuximab	mPFS:3.6m; mOS:11.6m	[38]
Antiangiogenic agents	VEGFR	Regorafenib	Phase I	mCRC	At least 4-line treatment	Regorafenib + cetuximab	PR:1/17; SD: 7/17	[39]

**Table 2:** Strategies to reverse resistance to anti-EGFR mAbs in preclinical studies

Therapy	Target	Agents	Species	Subpopulation	Treatment regimen	Efficiency	Reference
New anti-EGFR mAbs	EGFR ECD	GC1118	PDXs	*KRAS* mutation	GC1118 VS cetuximab	Sensitive VS insensitive	[40]
		MM-151	PDXs	*EGFR* G465E	MM-151 VS cetuximab/panitumumab	Sensitive VS insensitive	[41]
MEK inhibitor	MEK	Pimasertib	Cell	/	Pimasertib + cetuximab	Sensitive VS insensitive	[42]
ERBB2 mABs	ERBB2	4D5	Cell	/	4D5+ cetuximab VS cetuximab	Sensitive VS insensitive	[43]
		Trastuzumab	Cell	/	Trastuzumab + cetuximab VS cetuximab	Sensitive VS insensitive	[44]
PI3K inhibitor	PI3K	BKM120	Cell/nude mice	*KRAS* mutation	Cetuximab + BKM120 VS cetuximab VS BKM120	More effective	[45]
MET inhibitor	MET	Crizotinib	Cell	*KRAS* mutation	Crizotinib VS cetuximab	Sensitive VS insensitive	[46]
Metabolic regulators	AMPK	Metformin	Cell/ mice	*KRAS* mutation	Metformin+ cetuximab VS cetuximab	Sensitive VS insensitive	[47]
Metabolic regulators	Methylglyoxal	Carnosine	Cell/mice	*KRAS* mutation	Carnosine + cetuximab VS cetuximab	Sensitive VS insensitive	[48]
Metabolic regulators	BRAF	Simvastatin	Cell/mice	*KRAS* mutation	Simvastatin + cetuximab VS cetuximab	mean tumor volume: 20.2vs 49.4cm^3^	[49]
Metabolic regulators	Glutaminase 1	CB-839	Cell/mice	*KRAS* WT	CB-839 + cetuximab VS cetuximab	Sensitive VS insensitive	[50]
Metabolic regulators	RAF	L-ascorbic acid	Cell/mice	*KRAS* mutation	L-ascorbic acid + cetuximab VS cetuximab	Sensitive VS insensitive	[51]
Immunity therapy	NK cells	anti-CD137 mAb	Mice	*KRAS* WT/mutaion	anti-CD137 mAb + cetuximab	Tumor regression and prolonged survival	[52]
		UCB-NK	Cell	*EGFR* (+) *RAS* mutation	UCB-NK + cetuximab	Sensitive VS insensitive	[53]
Immunity therapy	T cells	BiTE	Cell	*RAS and RAF* mutation	BiTE+ cetuximab vs cetuximab	Sensitive VS insensitive	[54]
Immunity therapy	TLR9	IMO	Cell	*KRAS* mutation	IMO + cetuximab VS cetuximab	Sensitive VS insensitive	[55, 56]
Immunity therapy	CAFs	Regorafenib	Cell/ nude mice	Unselected	Regorafenib + cetuximab	Sensitive VS insensitive	[57]
		BLU9931	Cell	Unselected	BLU9931 + cetuximab VS cetuximab	Sensitive VS insensitive	[58]
Cytotoxic drugs	/	TAS-102	PDXs	/	TAS-102+Panitumumab	Response	[59]
Natural bioactive monomer	/	β-elemene	Cell / mice	*KRAS* mutation	β-elemene + cetuximab VS cetuximab	Tumor growth inhibition and less lymph node metastasis	[60]

### EGFR ligands and EGFR

EGFR is part of the EGFR tyrosine kinase family [61] and is activated by multiple ligands, such as EGF, TGF-α, HB-EGF, epiregulin (EREG) and amphiregulin (AREG) [62–64]. The expression of EGFR ligands in primary tumours is potentially related to anti-EGFR therapy efficiency [65, 66]. *KRAS* WT mCRC patients with higher expression of AREG and EREG seemed to obtain less survival benefit from cetuximab [64]. EGFR somatic sequence changes, including G465R, G465E, S468R and S492R, located at the extracellular domains (ECDs) of the EGFR-mAb interaction interface, confer resistance to cetuximab and panitumumab by preventing mAb binding [10, 67, 68]. In addition, R198/R200 methylation and mutation in the kinase domain of EGFR (V843I) correlated with disease progression in the presence of cetuximab [69].

Thus, the development of new mAbs that can bind to different or mutated EGFR ECDs is expected to improve the efficiency of anti-EGFR mAbs. MM-151, an oligoclonal antibody that binds multiple regions of the EGFR ECD, was confirmed to inhibit EGFR signalling and cell growth in a preclinical study and decrease mutations in circulating cell-free tumour DNA (ctDNA) of CRC patients [41]. Another FDA-approved EGFR antibody, necitumumab, can bind to S468R, the most common cetuximab-resistant variant of EGFR domain III [70]. Progression-free survival (PFS) and OS of patients taking necitumumab plus mFOLFOX6 were comparable to those of the cetuximab and FOLFOX regimens [21].

Considering the limitations of cetuximab and panitumumab in clinical use, it is necessary to generate more effective anti-EGFR antibodies. Sym004, a novel 1:1 mixture of two nonoverlapping anti-EGFR mAbs, showed significant advantages of abrogating EGFR ligand-induced phosphorylation and suppressing downstream signalling of all individual EGFR mutants both in cetuximab-resistant cell lines and in a tumour xenograft model [23, 71]. A multicentre, phase 2 clinical trial further confirmed that Sym004 improved the OS of anti-EGFR-refractory mCRC by 5.5 months [22]. In addition, GC1118 is a novel, fully humanized anti-EGFR IgG1 antibody that displays inhibitory effects against patient-derived xenografts from CRC tumours with a *KRAS* mutation [40], especially in those with elevated expression of high-affinity ligands [72].

### Compensatory feedback loop signalling

The RAS/RAF/MEK/ERK and PI3K/PTEN/AKT axes are the main downstream signalling pathways of EGFR. Upregulated receptor tyrosine kinases (RTKs), including ERBB2, MET and IGF-1R, activate the PI3K/AKT axis or reactivate the ERK pathway independently of EGFR [73–76]. Alterations in these pathways, such as gene mutation, gene amplification, gene loss and abnormal phosphorylation, are of great significance in primary and secondary resistance to anti-EGFR mAbs [11, 77]. Combining EGFR-targeted inhibitors with these targeted agents shows potential to reverse resistance to anti-EGFR mAbs.

#### RAS mutations and RAS regulators

RAS is a master element at the centre of EGFR signalling pathways [78]. Mutations within *RAS* put the RAS protein in a constitutively active state independent of upstream signals driven by growth factor receptor [79], leading to the failure of EGFR-targeted therapies. Mutations in *RAS* usually occur in *KRAS*, *NRAS* and *HRAS*, and the *KRAS* mutation is the most common of these genomic alterations, occurring in 40% of mCRC [9, 80]. Mutations in exons 2, 3 and 4 of *KRAS* and exons 2, 3, and 4 of *NRAS* are powerful predictors for cetuximab and panitumumab response in mCRC [81, 82]. However, codon 13 mutations (G13D) in *KRAS* do not predict nonresponse with complete accuracy [82]. Some missense and nonsense mutations at codons 20, 27, 30, or 31 have also been reported, whereas the function of these mutations on GTPase activity and the outcome of CRC still needs further exploration [83–85].

RAS was the first driver gene found, and effective RAS inhibitors have been investigated for over 30 years [86]. For example, sotorasib is a small molecule that selectively and irreversibly targets KRAS (G12C). Nevertheless, drugs again other *KRAS* mutations in codons 12, 13 and 61 still remain to be developed [87]. Therefore, it is important to find other therapies to improve the therapeutic outcome of these patients. In 2011, Wheeler and colleagues first reported that the addition of dasatinib to cetuximab showed a powerful antiproliferative effect on *KRAS* mutant cell lines compared to either agent alone in vitro and in vivo [88]. However, the other clinical study did not achieve the expected results. A phase IB/II study of 77 refractory CRC patients treated with dasatinib plus FOLFOX and cetuximab did not demonstrate meaningful clinical activity because the treatment did not fully inhibit the intracellular tyrosine kinase Src [24]. Notably, some untargeted agents displayed positive results in KRAS-mutated CRC cells. The combination of simvastatin and cetuximab suppressed BRAF activity and reduced the proliferation of *KRAS*-mutant cells [49]. Furthermore, Metformin reversed KRAS-induced resistance to the anti-EGFR antibody by activating AMP-activated protein kinase (AMPK) and inhibiting mTOR [47]. Jung revealed that resistance to cetuximab in CRC cells with *KRAS* mutations can be bypassed by L-ascorbic acid relying on a sodium-dependent vitamin C transporter 2 [51]. In addition, small chemical compounds such as KY7749 and methylglyoxal scavengers resensitize *KRAS*-mutated CRC cells to cetuximab in vivo [48, 89]. Despite not specifically targeting the RAS protein, these drugs add alternative methods to reverse resistance induced by *RAS*.

#### RAF mutations and RAF inhibitors

BRAF is a serine-threonine kinase just downstream of EGFR/KRAS that activates the MEK/extracellular signal-regulated kinase (ERK) signalling cascade through its phosphorylation and then promotes cancer cell proliferation [90]. *BRAF* mutation is a powerful biomarker of poor prognosis for mCRC patients receiving anti-EGFR mAbs [80, 91, 92]. The hotspot *BRAF* V600E mutation at codon 600 of exon 15 increases the activity of BRAF kinase by 130- to 700-fold [26, 93–95]. The prevalence of *BRAF* V600E mutations in mCRC is 8–10%, and they occur mutually exclusively with *KRAS* mutations [25, 96]. Some *BRAF* non V600E mutations were also reported, including D594G, G469A, L485F, L525R, Q524L and V600R located in the kinase domain. Non V600E mutations, other than Q524L, may also contribute to primary resistance to anti-EGFR mAbs [25, 96].

*BRAF* V600E mutations occur in various cancers, such as melanoma, non-small-cell lung cancer, breast cancer and CRC, and inhibitors targeting BRAF have demonstrated clinical benefit for these patients. Vemurafenib, a selective oral inhibitor of the *BRAF* V600 kinase, achieved an approximately 50% response and improved survival among metastatic melanoma patients with the *BRAF* V600E mutation [97]. Recently, many clinical trials have been conducted to evaluate EGFR therapeutic resistance with vemurafenib. In 2015, a pilot trial of combined vemurafenib and panitumumab in *BRAF*-mutant mCRC patients post chemotherapy reported that the treatment limited tumour progression and resulted in modest clinical activity [98]. However, a multicentric clinical study containing 27 CRC patients showed that vemurafenib alone or with cetuximab did not benefit CRC patients [99]. The next year, a phase IB study affirmed the value of vemurafenib again. This study demonstrated that triplets of vemurafenib, irinotecan, and cetuximab were well tolerated and exceeded tumour regression in refractory *BRAF*-mutated mCRC [100]. Of course, more clinical studies are needed to ensure that vemurafenib is efficacious.

Despite the confusing results of the vemurafenib studies, another BRAF inhibitor, encorafenib, has confirmed the feasibility of dual-targeted EGFR and BRAF treatment to increase the efficiency of anti-EGFR mAbs. The BEACON trial showed promising efficacy results with an objective response rate (ORR) of 48% (95% CI, 29.4–67.5%) among 29 patients in the study [27]. Within the randomized portion of the BEACON trial, the confirmed ORR for the triplet treatment was much better than that for the control (26% vs. 2%). The median OS was 9 months on the triplet regimen compared to 5.4 months in the control group (*P* < 0.0001, 75]. Based on the randomized, phase III BEACON trial, a combination of encorafenib and binimetinib (a MEK inhibitor) with cetuximab has been recommended as a second-line systemic therapy for *BRAF* V600E mutation CRC.

#### MEK activation and MEK inhibitors

MEK/ERK is the most important downstream cascade of the signalling pathways related to anti-EGFR mAbs. However, mutations of *RAS/RAF* induce constitutive activation of MEK to promote cell proliferation and survival. Encouragingly, combination treatment with MEK and EGFR inhibitors seems to be a possible strategy to overcome the multifaceted clonal heterogeneity in tumours [29, 101]. Additionally, there are already some small molecule MEK inhibitors under research. AS703026 (also known as pimasertib), AZD6244 (also known as selumetinib) and BAY86–9766 have a great ability to hinder the growth of mutant *KRAS* cells in vitro and in vivo by specifically suppressing the key target kinase ERK, which is downstream of MEK [30, 42]. MEK inhibitors were also confirmed to increase the tumour-suppressive effect of cetuximab. Selumetinib or pimasertib plus cetuximab enhanced antiproliferative and proapoptotic effects in cells resistant to cetuximab in vitro and in vivo [102, 103]. However, Misale found that in vitro and in vivo, the growth of resistant cells could not be hampered by MEK1/2 inhibitors alone; instead, the synergistic pharmacological blockade of EGFR and MEK induced drawn-out ERK inhibition and serious growth impairment of resistant tumour cells [43]. More importantly, the combination of selumetinib and cetuximab failed to achieve positive results in a clinical trial in refractory metastatic CRC patients with *KRAS* mutations [30, 101] To date, binimetinib is the only MEK inhibitor permitted by the Food and Drug Association for clinical use in mCRC. Clinical verification of the feasibility of MEK inhibitors to reverse EGFR therapeutic resistance is urgently required.

#### PI3K/AKT activation and PI3K/AKT inhibitors

Phosphor-EGFR is capable of initiating the PI3K/AKT/mTOR pathway, and *PI3K* mutation and aberrant AKT/mTOR activation promote resistance to anti-EGFR mAbs [104]. The most common mutations in *PIK3CA* are in exons 9 (68.5%) and 20 (20.4%), and they are detected in 10–18% of mCRC [11, 105]. Mutations in *PIK3CA* exon 20 were significantly associated with a worse outcome in *KRAS* WT mCRC patients treated with cetuximab, whereas *PIK3CA* exon 9 mutations had no effect on outcome in *KRAS* WT mCRC patients [11]. PTEN is a negative regulator in the PI3K/AKT pathway and was found in 20–40% of mCRC [106, 107]. Loss of PTEN protein results in long-term tumour growth by activating PI3K/AKT. Patients with PTEN-negative status showed a worse response rate and shorter progression-free survival (PFS) than those with PTEN-positive status [106, 108, 109]. *PI3K* gene mutation and PTEN protein loss are confirmed as novel biomarkers in mCRC patients treated with anti-EGFR mAbs [110].

Combinations of cetuximab and PI3K, AKT or mTOR inhibitors can profoundly control tumour growth in mCRC regardless of driver genotypes [32]. Specifically, PI3K inhibitors have been shown to greatly inhibit the growth of cancer in preclinical and clinical experiments. For example, the PI3K inhibitor XL147 was reported to inhibit the PI3K pathway with a 40–80% reduction in the phosphorylation of AKT and 4EBP1 in tumours and unexpectedly inhibited the MEK/ERK pathway in a phase I trial [111]. Then, in an investigation of the effects of XL147 on proliferation in a panel of tumour cell lines, Shapiro et al. revealed that XL147 was useful for PI3K mutation/amplification cell lines without KRAS/BRAF/PTEN mutation [112]. Another PI3K inhibitor, BKM120, was found to impede KRAS mutation-induced colorectal cancer growth both in vitro and in vivo, regardless of PI3K genotype [45]. Despite the ideal results of preclinical studies, clinical trials on the combination of PI3K inhibitors and EGFR-targeted agents are frustrating. PX-866 is a panisoform inhibitor of PI3K; however, the addition of PX-866 to cetuximab did not improve the PFS and OS of *KRAS* WT mCRC and caused greater toxicity in a phase II study [32]. Considering the lack of clinical trials on the combination of PI3K inhibitors with cetuximab or panitumumab, the application of PI3K inhibitors in enhancing the response to anti-EGFR mAbs remains unascertainable thus far.

#### ERBB2 amplification/mutations and ERBB2 inhibitors

A total of 2–7% of unselected CRCs were found to have ERBB2/mutations, and this number was much higher in KRAS wild-type cases (13.6%) and in *KRAS/NRAS/BRAF/PIK3CA* quadruple wild-type cases (36% )[13, 113, 114]. Bertotti et al. identified ERBB2 as a biomarker of resistance to anti-EGFR mAbs [113]. Importantly, amplification of ERBB2 was also enriched in nonresponsive *KRAS* WT mCRC [73]. ERBB2 activating mutations of S310F, L755S, V777L, V842I, and L866 increase MAPK phosphorylation and produce resistance to cetuximab and panitumumab [13].

The clinical use of ERBB2-targeted drugs, such as trastuzumab, pertuzumab and lapatinib, improved outcomes for breast cancer and colorectal cancer patients with *ERBB2* amplification [115, 116]. Dual-targeted therapy with EGFR and ERBB2 inhibition were found to restore sensitivity to cetuximab in vitro and in vivo. The monoclonal antibody 4D5 is an ERBB2 inhibitory antibody that shows antitumour function in an EGFR-dependent manner. The combination of the mAb 4D5 with cetuximab induced a significant decrease in proliferation in the EGFR-dependent colon cancer cell line and an actual regression of the tumours in xenografted mice [43]. Similarly, trastuzumab, the most common antibody for ERBB2, inhibits the growth of CRC cells when combined with cetuximab [44]. However, the pan ERBB kinase inhibitor neratinib plus cetuximab did not reach an objective response in anti-EGFR treatment refractory mCRC with quadruple-wild-type (*KRAS, NRAS, BRAF, PIK3CA*) in a phase II clinical trial [31]. In general, it will be important to investigate the efficiency of mAb 4D5 and trastuzumab in the clinic to confirm the value of anti-ERBB2 agents.

#### IGF-1R activation and anti-IGF-1R mAbs

The IGF-1/IGF-1R pathway plays a crucial role in CRC proliferation, differentiation, apoptosis, migration and angiogenesis. Hyperactivation of IGF-1R results in primary and secondary resistance to EGFR inhibition in RAS wild-type mCRC by upregulating the PI3K/AKT pathway [117, 118]. Analyses from two clinical trials confirmed that the coexpression of pIGF-1R and MMP-7 in *RAS* wild-type mCRC predicts worse OS after treatment with cetuximab [119].

Therefore, targeting both EGFR and IGF-1R may be a potential therapy for mCRC. Disappointingly, a trial showed that the combination of cetuximab and dalotuzumab or IMC-A12 did not improve the survival of mCRC resistant to cetuximab [35, 36].

#### MET amplification/activation and MET inhibitors

The MET signalling pathway is another compensatory feedback loop that mostly arises during the treatment of anti-EGFR mAbs. Phosphorylation of MET induces the activation of PI3K/AKT and RAS/RAF/MAPK cascades to rescue tumour cells from EGFR inhibitors [120]. Bardelli et al. highlight that *MET* amplification is related to acquired resistance to anti-EGFR therapy in tumours without *KRAS* mutations [12]. Moreover, the EGF ligands HGF and TGF-α can bind to MET and then increase the phosphorylation of MET and its downstream MAPK and AKT [121, 122]. Accordingly, the resistance function of MET was demonstrated by combining MET inhibitors and cetuxima b [123].

Therefore, the application of MET inhibitors has strong therapeutic potential in human cancers. The combination of MET inhibitors with anti-EGFR agents presents encouraging results in both preclinical and clinical studies. Tivantinib (ARQ 197), a selective, non-ATP-competitive inhibitor of c-MET, displayed tolerated toxicity and suggested some activity in previously treated mCRC when combined with cetuximab and chemotherapy [124]. Another phase II clinical study reported a positive outcome in mCRC patients who received tivantinib plus cetuximab. Forty-one patients with tumour progression on cetuximab or panitumumab treatment were enrolled in the study; the ORR was 9.8% (4/41), the median progression-free survival (mPFS) was 2.6 months (95% CI, 1.9–4.2 months), and the mOS was 9.2 months (95% CI, 7.1–15.1 months) [33]. Another small molecule c-MET inhibitor, crizotinib, has been shown to improve the efficiency of radiotherapy in cetuximab-resistant *KRAS* mutant CRC cell lines [46]. At the American Society of Clinical Oncology (ASCO) 2019, the phase II multicentre, multicohort GEOMETRY mono-1 clinical study showed that the combination of capmatinib (a c-MET inhibitor) with gefitinib (EGFR-TKI) had a good overall response rate in EGFR-TKI-resistant patients, particularly those with *MET*-amplified disease [34]. Furthermore, capmatinib plus cetuximab suggested that there were preliminary signs of activity in MET-positive mCRC patients who had progressive disease following anti-EGFR mAbs [125]. Suppression of MET will be an important target in overcoming resistance to anti-EGFR therapy.

### Microsatellite instability and immune checkpoint inhibitors

Microsatellite instability (MSI) caused by dysfunctional mismatch repair (dMMR) is detected in approximately 15% of all CRC and in nearly all cases with Lynch syndrome [126]. Microsatellite status and cetuximab efficiency is another area of interest. In the CALGB/SWOG 80405 study, patients with microsatellite instability-high (MSI-H) tumours showed worse OS in the cetuximab arm than in the bevacizumab arm [127]. MSI may interact with oncogenic drivers such as *BRAF* and *ERBB2* to promote cetuximab resistance. *BRAF* V600E occurs in 40% of sporadic MSI-H CRCs and is typically genetically seen subsequent to *hMLH1* hypermethylation [128]. In addition, other hotspot mutations in *KRAS*, *PIK3CA* and *ERBB2* were identified in *BRAF* WT MSI CRC patients [129]. It has been proven that *hMLH1* deficiency plays a role in cetuximab resistance by increasing the expression level of ERBB2 and downstream PI3K/AKT signalling [130]. Although we believe that mismatch repair genes may partly modulate the expression of oncogenic drivers, the mechanism remains largely unclear and a worthwhile focus for further research.

EGFR-targeted treatment increased the infiltration of cytotoxic immune cells and the expression of the PD-L1 immune checkpoint, which may be a potential method to treat cetuximab-resistant CRCs with immunotherapy [19]. In addition, NK cell-mediated ADCC activated by cetuximab triggers immunogenic death of tumour cells, thereby increasing the antitumour activity of immunotherapy [131]. The phase II CAVE mCRC trial demonstrated that rechallenge avelumab (anti-PD-L1) plus cetuximab resulted in a mPFS of 3.6 months and a median OS (mOS) of 11.6 months in a *RAS* WT mCRC population who developed acquired resistance to anti-EGFR drugs [38]. The ongoing AVETUXIRI trial investigates the efficiency of avelumab combined with cetuximab and irinotecan for refractory mCRC patients with microsatellite stability. The current study data has shown that encouraging results of DCR, PFS and OS were observed in both the RAS MT and RAS WT cohorts [132]. The combination of immune checkpoint inhibitors with anti-EGFR mAbs may bring great breakthroughs to overcome resistance to anti-EGFR drugs and improve the outcome of mCRC regardless of the status of *RAS*.

### Metabolic remodelling and regulators

Alterations in cellular metabolism are essential for rapid tumour proliferation and affect the sensitivity of cancer cells to various drugs [133]. Anti-EGFR treatment causes metabolic rewiring in CRC patients, which makes it possible to increase anti-EGFR mAb efficiency by adding metabolism regulators.

Abnormal glycometabolism reduces the efficiency of anti-EGFR therapy. High glycolytic metabolism regulated by TRAP1 was involved in resistance to EGFR mAbs [15]. Sirt5-positive CRCs develop cetuximab resistance due to an elevated succinate-to-ketoglutarate (αKG) ratio, which inhibits αKG-dependent dioxygenases [134]. Sodium glucose transporter 2 (SGLT2) can ensure glucose entry into cells and is highly expressed in the majority of cancer cells. The SGLT2 inhibitor dapagliflozin combined with cetuximab dramatically reduced carcinoembryonic antigen (CEA) and substantial shrinkage of metastatic tumour lesions [37]. The methylglyoxal scavenger carnosine was confirmed to resensitize *KRAS*-mutated colorectal tumours to cetuximab in vivo [48]. AMPK activity was consistent with the sensitivity of anti-EGFR mAbs, and metformin overcame *KRAS*-induced resistance to anti-EGFR antibodies by regulating AMPK/mTOR/Mcl-1 (myeloid cell leukaemia 1) in vivo and in vitro [47]. Fatty acid metabolism displayed strong antiapoptotic effects in cetuximab-nonresponders [135]. Inhibition of lipid synthesis or decomposition with simvastatin or glutaminase 1 inhibitor CB-839 significantly reduced tumour growth of CRC under cetuximab treatment [49, 50]. In addition, vitamin D deficiency has a negative impact on cetuximab-induced ADCC. Supplementation with vitamin D in vitamin-deficient/insufficient CRC cells has been suggested to improve cetuximab-induced ADCC in CRC cell lines [136]. Resistance to cetuximab in mutant *KRAS* CRC patients can be reversed by L-ascorbic acid by reducing RAF/ERK activity in an SVCT-2-dependent manner [51].

### Others

Autophagy and cancer stem cells (CSCs) also contribute to resistance to EGFR target therapy [18, 137]. Treatment with anti-EGFR agents results in dysregulation of autophagy [138]. Increased levels of autophagy-related proteins such as Beclin-1 and LC3 were observed in cetuximab-treated patients [139, 140]. Inhibition of autophagy by chloroquine and 3-methyladenine sensitizes cancer cells to cetuximab [138, 139]. However, blocking general autophagy might greatly affect normal cell growth. Therefore, developing specific autophagy inhibitors that target tumour cells is crucial.

CSCs possess genetic determinants for the EGFR therapeutic response and are primarily supported by a network of pluripotency transcription factors (PTFs). Single-nucleotide polymorphisms of PTFs were significantly associated with PFS of the cetuximab cohort in the FIRE-3 trial [141]. The property of CSCs to EMT is a core transcriptional network to predict the efficacy of EGFR-targeted therapy in *KRAS* WT CRC [142]. Inhibition of EMT is of great interest for reversing EGFR therapeutic resistance. β-Elemene, a bioactive monomer isolated from the Chinese herb curcumae rhizoma, has been shown to induce ferroptosis and reduce EMT to increase cetuximab activity in *RAS*-mutated CRC cells [60]. Furthermore, cytotoxic drugs and natural bioactive monomers were confirmed to overcome resistance to EGFR-targeted drugs. TAS-102 is a novel chemotherapeutic agent that contains a thymidine phosphorylase inhibitor, tipiracil hydrochloride, and a cytotoxic thymidine analogue, trifluridine, which has been approved for the treatment of mCRC. Panitumumab/TAS-102 cotreatment showed additive antiproliferative effects in LIM1215 CRC cells in vitro and in vivo [59].

## Extrinsic mechanisms of resistance to targeted therapy and related strategies

**Fig. 2:**
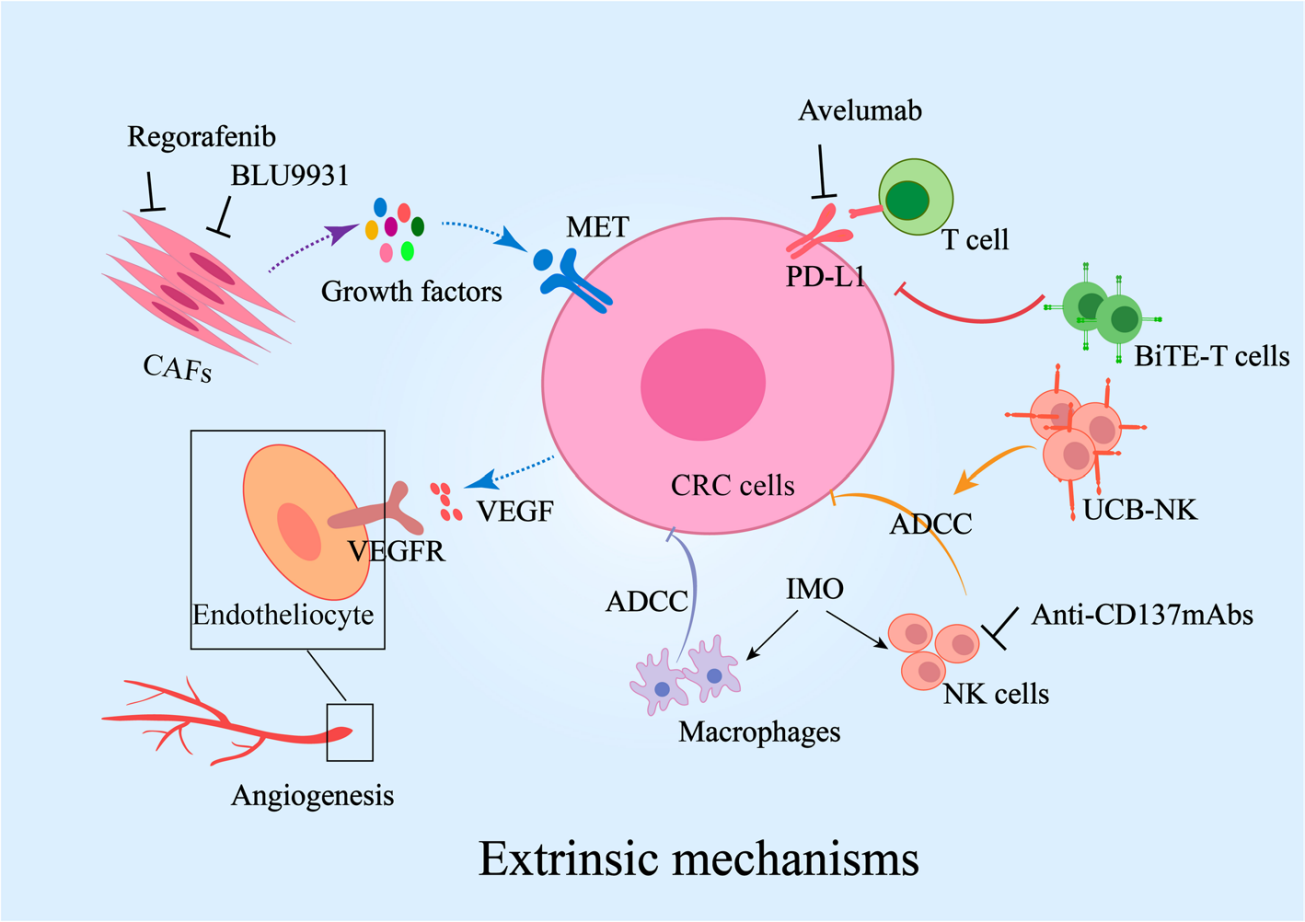
Extrinsic mechanisms of resistance to anti-EGFR mAbs in metastatic colorectal cancer. Tumour microenvironment plasticity confers resistance to EGFR-targeted therapy. Cetuximab and panitumumab suppress tumours through ADCC mediated by NK cells and macrophages. Dysfunction of NK cells and macrophages with lower ADCC impairs the suppression of EGFR-targeted therapy in cancer. Reduced density of effector T cells and increased PD-L1 expression in cancer cells also promote survival from cancer. CAFs promote resistance to targeted therapy by secreting growth factors that activate the RAS or MET pathway. Abnormal angiogenesis always predicts poor response to anti-EGFR mAbs. Therapies focused on the microenvironment are also shown in the figure. Abbreviations: CAFs, cancer-associated fibroblasts; NK cells, natural killer cells; ADCC, antibody-dependent cellular cytotoxicity; PD-1, programmed death 1; PD-L1, programmed death ligand 1. VEGF, vascular endothelial growth factor; VEGFR, vascular endothelial growth factor receptor

Microenvironmental plasticity dramatically affected by EGFR inhibition is as powerful of a driver of drug resistance as genetic alterations [18] (Fig. 2). Dysfunction of immune cells, abnormal infiltration of cancer-associated fibroblasts (CAFs) and angiogenesis impair EGFR therapeutic efficiency. Strategies to remodel the tumour microenvironment are part of a larger goal to increase the efficiency of anti-EGFR mAbs. These strategies include (i) modification or activation of NK cells and T cells, (ii) suppression of CAFs, and (iii) inhibition of angiogenesis (Tables 1 and 2).

### Immune cells and agents

Antibody-dependent cellular cytotoxicity (ADCC) mediated through Fc receptors (FcγRs) on immune cells is one of the proposed antitumour mechanisms of anti-EGFR mAbs [20]. Cetuximab-treated patients with FcγRIIa-131R and/or FcγIIIa-158F genotypes had shorter PFS than 131H/H and/or 158 V/V carriers [143]. When exposed to cetuximab in CRC cell lines, human NK cells substantially increase the expression of the costimulatory molecule CD137 (4-1BB )[144]. The combination of cetuximab with anti-CD137 mAb administration was synergistic and resulted in complete tumour resolution and prolonged survival, which were dependent on the participation of NK cells [52]. IL-2 and IL-15 cooperate with cetuximab to stimulate NK cells and improve cytotoxic functionality [145]. Another preclinical study using a mouse model reported that umbilical cord blood stem cell-derived NK (UCB-NK) cells increased antitumour cytotoxicity against CRC regardless of the status of *EGFR* and *RAS* [53]. Neither cetuximab nor panitumumab can engage T cells when T cells lack Fcγ receptors, which serve as targets for modifying T cells to enhance the ADCC activity of anti-EGFR agents [54]. T cell-engaging BiTE antibodies targeting the binding domains of cetuximab and panitumumab transiently connect T cells with cancer cells to initiate redirected target cell lysis. Then, they showed that cetuximab-based BiTE antibody mediated potent redirected lysis of *KRAS*- and *BRAF*-mutated CRC lines in vitro and prevented the growth of tumours from xenografts [54]. Toll-like receptor 9 (TLR9) is expressed in various immune cells, such as macrophages, NK cells, B lymphocytes and plasmacytoid dendritic cells [146, 147]. Toll-like receptor 9 (TLR9) activation causes antitumour activity by interfering with cancer proliferation and angiogenesis [148]. IMO is a novel second-generation, modified, immunomodulatory TLR9 agonist and was proven to synergistically inhibit tumour growth by improving the ADCC activity of cetuximab in a cetuximab-resistant colorectal cancer line and a mouse model regardless of KRAS genotype [55, 56].

### CAFs and inhibitors

Cancer-associated fibroblasts (CAFs) are believed to play a vital role in promoting tumour metastasis and drug resistance by secreting mitogenic growth factors, including FGF1, FGF2, HGF, TGF-β1 and TGF-β 2 [19]. Luraghi et al. reported that HGF can bind to MET receptors and activate MAPK and AKT to induce cetuximab resistance in vitro [75]. The dual inhibition of FGFR and EGFR may be a practical strategy to reverse resistance to anti-EGFR mAbs. The combination of BLU9931,an FGFR4 inhibitor, with cetuximab presented profound antitumour activity compared to cetuximab alone [57]. Regorafenib, a multikinase inhibitor targeting FGFR, VEGF and PDGFR-β, was found to overcome cetuximab resistance in GEO-CR and SW48-CR cells in vitro and in vivo [58].

### Angiogenesis and inhibitors

Inhibition of angiogenesis is also one of the mechanisms of cetuximab action. Treatment with cetuximab reduced the expression of vascular endothelial growth factor (VEGF), and a high level of VEGF under cetuximab treatment was associated with a lower response rate and shorter PFS in mCRC [149, 150]. VEGF is one of the most significant angiogenetic factors, and it contributes to cancer prognosis and metastasis. Therefore, it is worth exploring the feasibility of dual-targeted VEGF and EGFR in colorectal cancer. Combination treatment with anti-VEGF and anti-EGFR antibodies demonstrated synergistic activity in vitro, and tumour growth and angiogenesis were strongly suppressed in an in vivo xenograft mouse model [151]. However, in another study, the use of bevacizumab and cetuximab together did not have a greater increase in apoptotic tumour cell death compared to either drug alone [152]. Recently, small molecule inhibitors targeting VEGF have presented the potential to increase the efficiency of anti-EGFR therapy. Pazopanib, a multitargeted tyrosine kinase inhibitor, combined with irinotecan and cetuximab showed manageable safety and feasibility in refractory mCRC [153]. The combination of the anti-EGFR antibody cetuximab and the multikinase VEGF inhibitor regorafenib overcame intrinsic and acquired resistance in mCRC. Eight of 17 mCRC patients, who all were previously receiving anti-VEGF and anti-EGFR therapy, showed clinical benefit from cetuximab and regorafenib, including partial response in 1 patient and stable disease in 7 patients [39]. Dual-targeting of VEGF and EGFR seems to be an effective choice for mCRC patients receiving multiline treatment.

## Conclusions and future directions

**Fig. 3:**
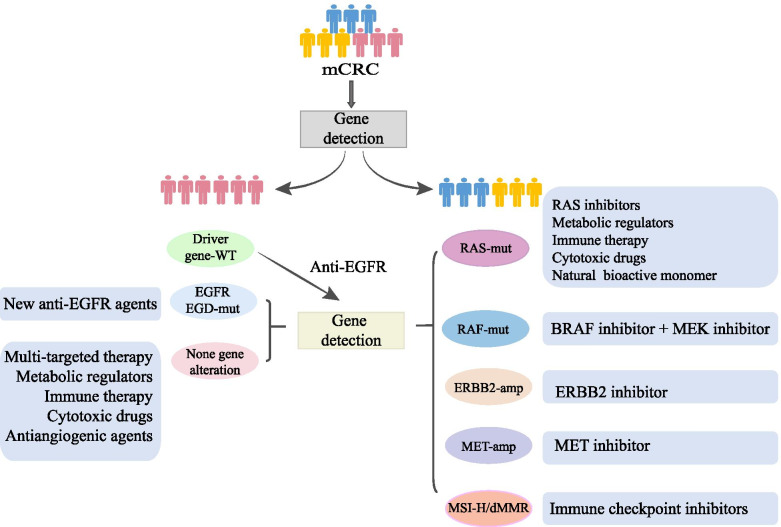
Strategies to increase anti-EGFR therapy efficiency in different subtypes of mCRC. Biomarker analysis should be conducted before treatment for mCRC. For patients with disease progression on anti-EGFR therapy, biomarker analysis is still recommended. For mCRC with driver gene alterations, there are some therapies to increase anti-EGFR efficiency. In RAS-mut mCRC, the selected therapies include a combination of RAS inhibitors and anti-EGFR agents, metabolic regulators, immune therapy, cytotoxic drugs and natural bioactive monomers. In RAF-mut mCRC, the main therapy is a BRAF inhibitor. In ERBB2-amp mCRC, ERBB2 inhibitors can be used to promote the antiproliferation of anti-EGFR. In MET-amp mCRC, combined therapy with MET inhibitors and anti-EGFR mAbs was confirmed to be effective. In mCRC with EGFR ECD-mut, new anti-EGFR agents are preferred. In mCRC with no driver gene alteration, multitargeted therapies, metabolic regulators, immune therapy, cytotoxic drugs and antiangiogenic agents can be used with anti-EGFR. Abbreviations: mCRC, metastatic colorectal cancer; EGFR, epidermal growth factor receptor; ERBB2, human epidermal growth factor receptor 2; MET, tyrosine-protein kinase Met; MSI-H, microsatellite instability; dMMR, dysfunctional mismatch repair; PD-1/PD-L1, programmed death-1/programmed death ligand 1; ECD, extracellular domain; WT, wild type; mut, mutation

Heterogeneity and adaptive alterations promote resistance to anti-EGFR targeted therapy and are strongly associated with the clinical outcome of colorectal cancer (Fig. 3). *RAS/BRAF/MEK* mutations downstream of the EGFR pathway and *ERBB2/MET/IGFR/PI3K* mutations or amplifications bypassing EGFR are strong biomarkers to predict the efficiency of anti-EGFR mAbs. It is of great importance to ascertain the molecular subtypes in mCRC before treatment. Advances in gene detection methods such as ctDNA, liquid biopsy and exosome DNA sequencing make molecular subtyping feasible. By identifying similarities and differences among tumour subtypes, the use of precision medicine results in greater cancer eradication and better patient care. For subpopulations with driver-gene mutations, combination therapies of different targeted inhibitors make great strides in overcoming resistance to anti-EGFR mAbs. Combining EGFR targeted therapy with inhibitors of BRAF, MET and MEK produces expected results in clinical trials. It is recommended to use encorafenib, binimetinib and cetuximab in the second-line treatment of mCRC. More clinical studies are needed to ensure the effectiveness of MEK inhibitors. In addition, the new generation of anti-EGFR monoclonal antibodies and cytotoxic agents is promising to achieve better outcomes, but further research is needed before clinical application.

In this review, we provide new insight into EGFR therapeutic resistance in the tumour microenvironment (TME) and summarize current agents for the TME. The TME, including the immune microenvironment and vascular microenvironment, facilitates tumour growth and metastasis. The ADCC activity of anti-EGFR mAbs mediated by NK cells, T cells and macrophages is one of the antitumour mechanisms targeted by cetuximab and panitumumab. The strong effect of cetuximab on the immune landscape dramatically changes immune infiltrates. Thus, more effective immunotherapies are anticipated to regress the growth and metastasis of tumours. Some antibodies or inhibitors constructed to bind FcγR or TLR9 to stimulate ADCC mediated by NK cells, T cells and macrophages present significant antitumour activity in cell lines and mouse models. Dual-targeted VEGF and EGFR treatments show exciting results in multiline-treated mCRC patients, providing a chance for improved outcomes in refractory patients. Notably, anti-EGFR therapy especially enhances the expression of PD-L1 on tumours and the infiltration of CD8^+^ T cells. Therefore, this feature may expand indications of immune checkpoint inhibitors in CRC. Treatment with immune checkpoint inhibitors either along with anti-EGFR mAbs or later is a promising therapy for mCRC.

In summary, the recognition of resistance to EGFR-targeted therapy has progressed from driver genes to nongenetic alterations. Different therapies that reverse EGFR therapeutic resistance demonstrate potential in preclinical and clinical trials. These treatments show promise in taking a giant step towards overcoming EGFR therapeutic resistance.

## Data Availability

All the data obtained and/or analyzed during the current study were available from the corresponding authors on reasonable request.

## Abbreviations

EGFR: Epidermal growth factor receptor
WT: Wild type
mAbs: Monoclonal antibodies
mCRC: Metastatic colorectal cancer
CRC: Colorectal cancer
PFS: Progression-free survival
OS: Overall survival
mPFS: median progression-free survival
mOS: median overall survival
ORR: Objective response rate
TKI: Tyrosine kinase inhibitors
ECD: Extracellular domain
ERK: Extracellular signal-regulated kinase
RTKs: Receptor tyrosine kinases
MSI: Microsatellite instability
dMMR: Dysfunctional mismatch repair
CSCs: Cancer stem cells
ADCC: Antibody-dependent cellular cytotoxicity
FcγRs: Fc receptors
CAFs: Cancer-associated fibroblasts
AMPK: AMP-activated protein kinase
ASCO: American Society of Clinical Oncology
SGLT2: Sodium glucose transporter 2
GLS1: Glutaminase1
TLR9: Toll-like receptor 9
FGFR: Fibroblast growth factor receptors
ctDNA: circulating cell-free tumor DNA
αKG: a-ketoglutarate
PTFs: Pluripotency transcription factors
VEGF: Vascular endothelial growth factor
VEGFR: Vascular endothelial growth factor receptor
